# An acute presentation of Chronic Thromboembolic Pulmonary Hypertension complicated by cerebellar infarction

**DOI:** 10.1016/j.rmcr.2023.101931

**Published:** 2023-10-18

**Authors:** M. Gleeson, J. Murray, S. Gaine

**Affiliations:** a Department of Respiratory Medicine, Mater Misericordiae University Hospital, Dublin 7, Ireland; b Department of Radiology, Mater Misericordiae University Hospital, Dublin 7, Ireland

## Abstract

Persistent symptoms and features of pulmonary hypertension in a patient with pulmonary embolism suggests chronic thromboembolic pulmonary hypertension (CTEPH) which can be associated with significant morbidity and mortality. A high level of clinical suspicion, including addressing risk factors for recurrent or cgronic thromboemboli and appropriate anticoagulation is required. We present a rare case of a young man presenting late with pulmonary embolism and features consistent with CTEPH complicated by a cerebrovascular event.

## 1 Introduction

Pulmonary hypertension (PH) is defined as an abnormal elevation of pressure in the pulmonary circulation. Regardless of the underlying aetiology, a pre-capillary mean pulmonary arterial pressure above 20 mmHg is considered abnormal [1]. Chronic Thromboembolic Pulmonary Hypertension (CTEPH), considered a small vessel vasculopathy, occurs in patients due to chronic pulmonary artery obstruction and is estimated to occur in 3.8% of patients following acute Pulmonary Embolism [2]. If untreated progressive right ventricular dysfunction and ultimately right heart failure can result in significant morbidity and mortality.

## 2 Case report

**Fig. 1:**
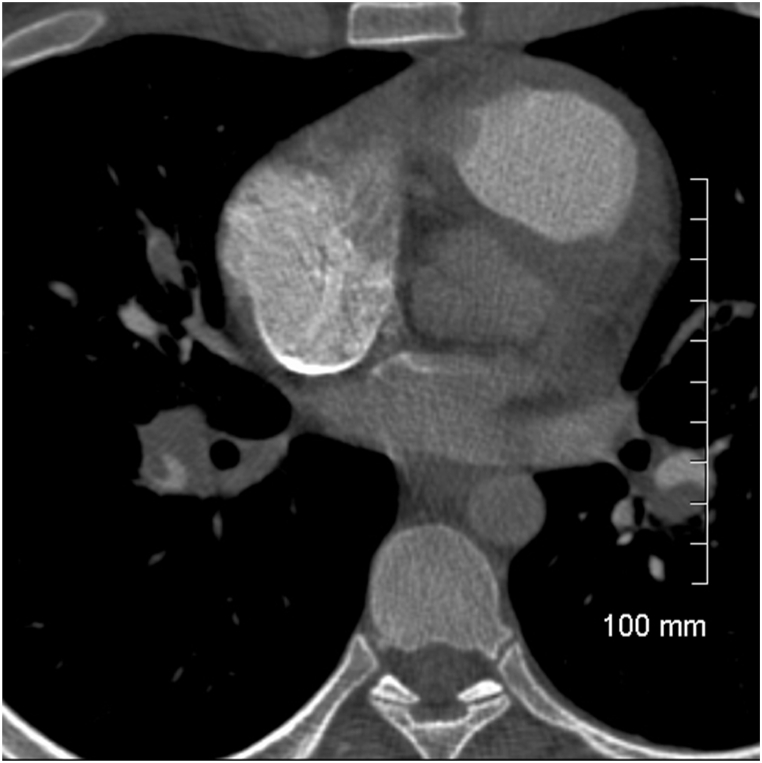
CT pulmonary angiogram demonstrating bilateral pulmonary emboli with contrast present in the left atrium suggesting PFO.

**Fig. 2:**
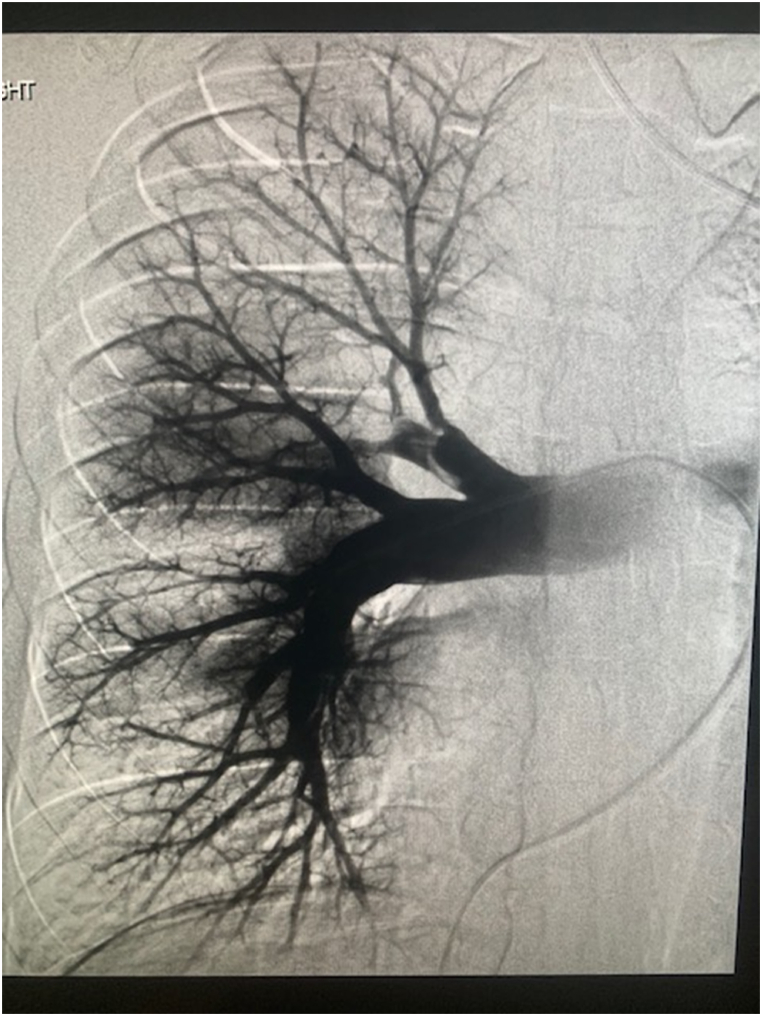
Pulmonary angiogram demonstrating pouch-like ending of pulmonary artery segments with pre stenotic dilation of the vasculature.

A previously well 24 year old male presented with progressive dyspnoea on exertion despite repeated treatment in the community for lower respiratory tract infection over the two months prior. Computed Tomography Pulmonary Angiogram (CTPA) identified bilateral pulmonary emboli with a dilated pulmonary artery and evidence of right heart strain (Fig. 1). Laboratory investigations revealed an elevated level of βeta_2_-Glycoprotein (B2 GP1) of 46IU/ml, suggestive of anti-phospholipid syndrome (APS). Despite theraputic anticoagulation he had a persistent oxygen requirement and remained symptomatic during his admission. Echocardiogram demonstrated features consistent with pulmonary hypertension with a RVSP of 55 mmHg (RV BD 5.3cm, TAPSE 1.0cm S′0.08m/s, TRVmax 3.7m/s), septal flattening and consequently reduced ejection fraction of 35–40%. Further investigation with invasive pulmonary angiogram demonstrated pulmonary artery stenosis, occlusion and wedge-shaped perfusion defects (Fig. 2). Immediately post-procedure he developed acute onset diplopia with an intranuclear opthalmoplegia on examination. While the neurological deficit was transient a subsequent MRI Brain demonstrated a 3mm punctate focus of increased signal hyperintensity within the right cerebellar hemisphere 48 hours later, consistent with an acute infarct. Restrospective review of his CTPA revealed a patent foramen ovale (PFO) (Fig. 1) and this was later confirmed with a bubble study and *trans*-oesophageal echocardiogram. He was commenced on Low Molecular Weight Heparin (LMWH), pulmonary vasodilator Riociguat and discharged home with long term oxygen.

Repeat thrombophilia screen confirmed APS with persistently raised beta 2 GP1 of 46, anticardiolipin antibodies (aCL) IgG of 133IU/ml and a strongly positive lupus anticoagulant at the nadir of his LMWH. At this point he was transitioned to Warfarin for lifelong anticoagulation. He underwent a successful pulmonary endartectomy (PEA) and remained symptom free at follow up.

## 3 Discussion

Pulmonary embolism typically presents with acute onset dyspnoea, chest pain, pre-syncope and less commonly syncope and haemoptysis [3]. Its presentation can often however be asymptomatic as an incidental finding or more insidious in nature. All clinicians dealing with pulmonary embolism should determine if it represents acute, subacute or chronic thromboembolic disease and have a high level of suspicion for pre-existing CTPEH if any atypical features present, clinically or radiologically as treatment and prognosis differ greatly [4,5].

Echocardiogram can highlight features of PH while a Ventilation-perfusion (V/Q) scan can confirm one or more segmental or larger mismatch perfusion defects but formal pulmonary angiography at least three months after initiation of appropriate anticoagulation is the gold standard for diagnosis [6]. Conventional pulmonary angiography can map the site and extent of disease and determine suitability for surgical intervention. A diagnosis of CTEPH usually requires right heart catheterisation to measure the pressures within the pulmonary circulation however, a decision was made to postpone this for our patient in the acute setting due the high risk of thrombotic events.

Thrombophilia screening is included in the work up for pulmonary embolism, although less than 10% of CTEPH patients have a provoking thrombophilia. Antiphospholipid syndrome can be suggested by positive anticardiolipin antibodies, lupus anticoagulants, and anti-β_2_-glycoprotein I antibodies, the mechanism by which they lead to a hypercoagulable state remains unclear however and repeat testing three months later is required for confirmation [7]. A positive lupus anticoagulant (LA) is the most commonly associated with recurrent thrombosis, however patients with triple positive screens; LA, aCL and B2GP1, are at the highest thrombotic risk [8].

Complications of pulmonary angiography include nephrotoxicity, cardiac arrythmias and in rare cases vascular injury, cardiac perforation and cardiac arrest [9]. Stroke has only been reported in one other case report as a complication of instrumentation [10]. Patent foramen ovale, a congential cardiac lesion, persists in 25–30% of adults [11]. The presence of an intracardiac defect creates a small left to right shunt under normal physiologic conditions. In the setting of high right sided pressures, as a result of an acute pulmonary embolism or by extension chronic pulmonary hypertension, a significant right to left shunt can develop. In this context intracardiac defects such as a PFO are associated with cryptogenic stroke in patients with venous thromboembolsim via paradoxical embolism [12]. Its significance is evident in our case with passage of thrombus from venous to arterial systems manifesting here as cerebral infaction. Given the transient nature of his symptoms no acute intervention with thrombolysis was required and there was no residual neurological deficit. PFO closure must be considered especially in patients where there remains a thromboembolic risk given the probable diagnosis of APS. In our case the PFO was considered small and closure was not indicated with the primary focus on treating the severe pulmonary hypertension in the first instance.

Medical treatment options for CTEPH focus on pulmonary vasodilators and remodelling agents to lower the pulmonary vascular resistance and pulmonary arterial pressure, improving symptoms such as exercise tolerance and oxygenation. Agents such as riociguat, a soluable guanylate cyclase, stimulator are limited to those with inoperable disease or with recurrent or persistent disease despite pulmonary endartectomy (PEA) [13]. When it comes to curative treatment surgery is the therapy of choice, especially when the bulk of the clot burden is in the proximal vessels. Our patient was referred to a specialist centre for consideration of PEA. In severe cases where surgery is not a viable option, Balloon Pulmonary Angioplasty (BPA) has been shown to significantly improve pulmonary arterial pressure [14], or alternatively patients can be referred for heart-lung transplant [15].

This case describes a rare series of events occurring following pulmonary emboli, highlighting the associated morbidity associated. It emphasises the importance of correct application of diagnostic tests in order to reduce any delay in diagnosis, identifying risk factors and initiating appropriate treatment for pulmonary embolism, reducing the risk of recurrence or progression to CTEPH.

## Declaration of competing interest

No conflict.
